# Case Report: SS18::POU5F1-fused sarcoma with Flexner-Wintersteiner rosettes: a clinicopathological analysis of a rare tumor in the submandibular gland

**DOI:** 10.3389/fonc.2026.1768229

**Published:** 2026-03-26

**Authors:** Zhaorong Niu, Guixiang Xiao, Yu Guo, Qin Xia, Jun Fan, Jun He, Danju Luo, Xiu Nie

**Affiliations:** 1 Pathology Department, Union Hospital, Tongji Medical College, Huazhong University of Science and Technology, Wuhan, China; 2 Department of Pathology, First People’s Hospital of Yibin City, Sichuan, Yibin, China

**Keywords:** Flexner-Wintersteiner rosettes, gene fusion, neural differentiation, SS18::POU5F1, submandibular gland

## Abstract

SS18::POU5F1-fused sarcoma is an exceedingly rare malignant tumor, with only isolated case reports documented to date. We present the first reported case of SS18::POU5F1 fusion sarcoma occurring in the left submandibular gland of a middle-aged male patient. The tumor exhibited multiphasic differentiation, including epithelioid cells and undifferentiated round cell components, with focal evidence of neural differentiation and Flexner–Wintersteiner rosettes. It was characterized by the presence of a SS18::POU5F1 gene fusion. We provide a detailed description of the clinical features, histomorphology, immunophenotype, molecular characteristics, treatment, and prognosis of this case, discuss its differential diagnosis and potential pathogenesis, and include a comprehensive review of the relevant literature.

## Introduction

With the rapid advancement of molecular pathology, significant progress has been made in recent years in understanding the molecular pathogenesis of undifferentiated small round cell tumors. However, certain rare cases remain difficult to classify. SS18::POU5F1-fused sarcoma is an exceedingly rare malignant tumor, with only isolated case reports documented in the literature to date (1–5). This tumor is characterized by epithelioid or undifferentiated round cell morphology and the presence of a distinct SS18::POU5F1 gene fusion.

This article presents the first reported case of SS18::POU5F1-fused sarcoma arising in the submandibular gland region. The tumor exhibited both epithelioid and undifferentiated round cell morphology, accompanied by neural differentiation and Flexner–Wintersteiner rosettes. Next-generation sequencing (NGS) confirmed the presence of the SS18::POU5F1 gene fusion.

We describe the clinicopathological features, molecular characteristics, treatment, and prognosis of this tumor, discuss its differential diagnosis and potential pathogenesis, and provide a comprehensive review of the existing literature on SS18::POU5F1-fused sarcoma to enhance understanding of this rare entity.

## Case presentation

**Figure 1:**
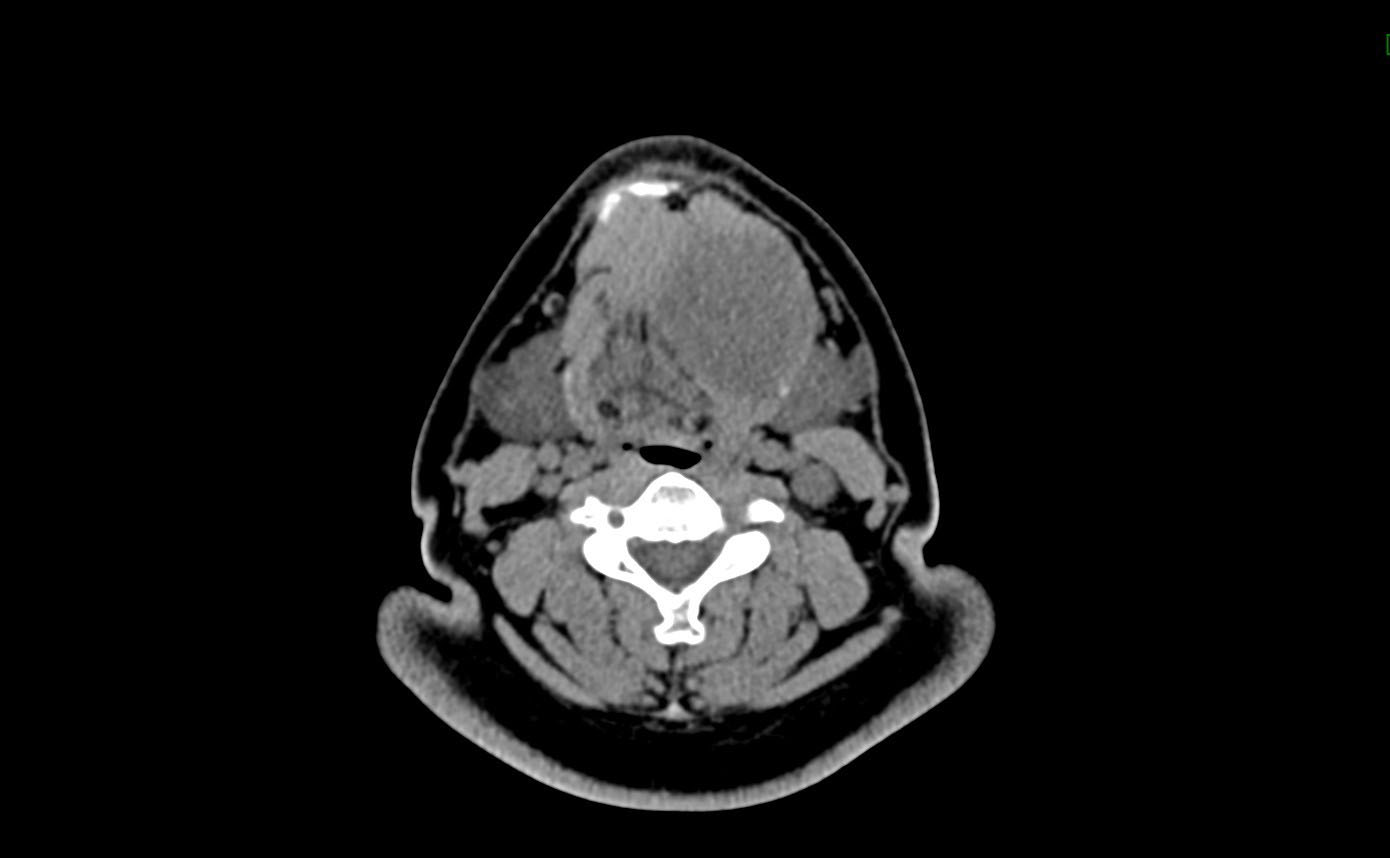
CT scan reveals a mass-like soft tissue density shadow in the left submental region, measuring approximately 53×50×49 mm.

A 38-year-old male patient was admitted on April 1, 2025, with a two-week history of a left submandibular mass. No significant past medical history was reported. Physical examination revealed a palpable, firm mass with reduced mobility and no obvious tenderness. Neck ultrasonography identified a heterogeneous lesion in the subcutaneous soft tissue layer of the left submandibular gland region. An unenhanced CT scan with three-dimensional reconstruction demonstrated a soft-tissue-density mass in the left submental region, measuring approximately 53 mm × 50 mm × 49 mm, situated lateral to the genioglossus muscle and involving the left mylohyoid muscle (**Figure 1**). The lesion exhibited an average attenuation value of 35 Hounsfield units. Multiple enlarged lymph nodes were observed in the submental, left submandibular, and left cervical regions, with the largest measuring approximately 18 mm × 27 mm. To further characterize the nature of the mass, a deep excision of the left neck mass was performed on April 3, 2025.

**Figure 2:**
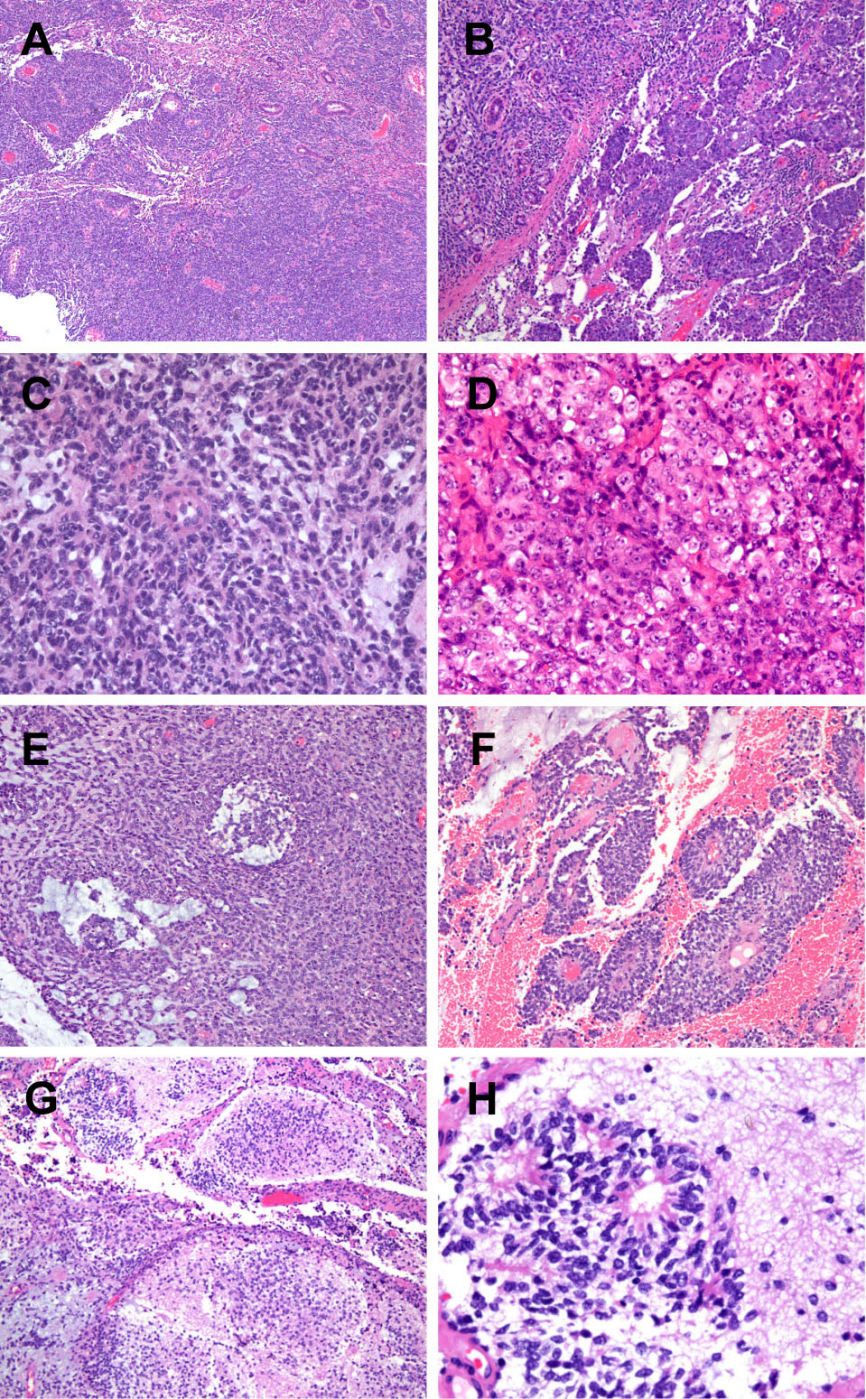
**(A)** The tumor exhibits solid nested-sheet growth patterns and is highly vascularized, with residual salivary gland tissue visible within; **(B)** Tumor cells demonstrate two distinct morphologies: poorly differentiated small round cells and epithelioid cells, with a clear demarcation between the two populations; **(C)** High-power view of the small round cells shows scant cytoplasm and round nuclei, with occasional irregular nuclear contours, fine chromatin, and small nucleoli; **(D)** High-power view of the epithelioid cells reveals abundant cytoplasm, relatively large nuclei, and prominent nucleoli; **(E, F)** Focal stromal myxoid changes and perivascular pseudopapillary structures are observed; **(G, H)** Areas of neural differentiation display a neuropil-like background and Flexner-Wintersteiner rosettes.

**Figure 3:**
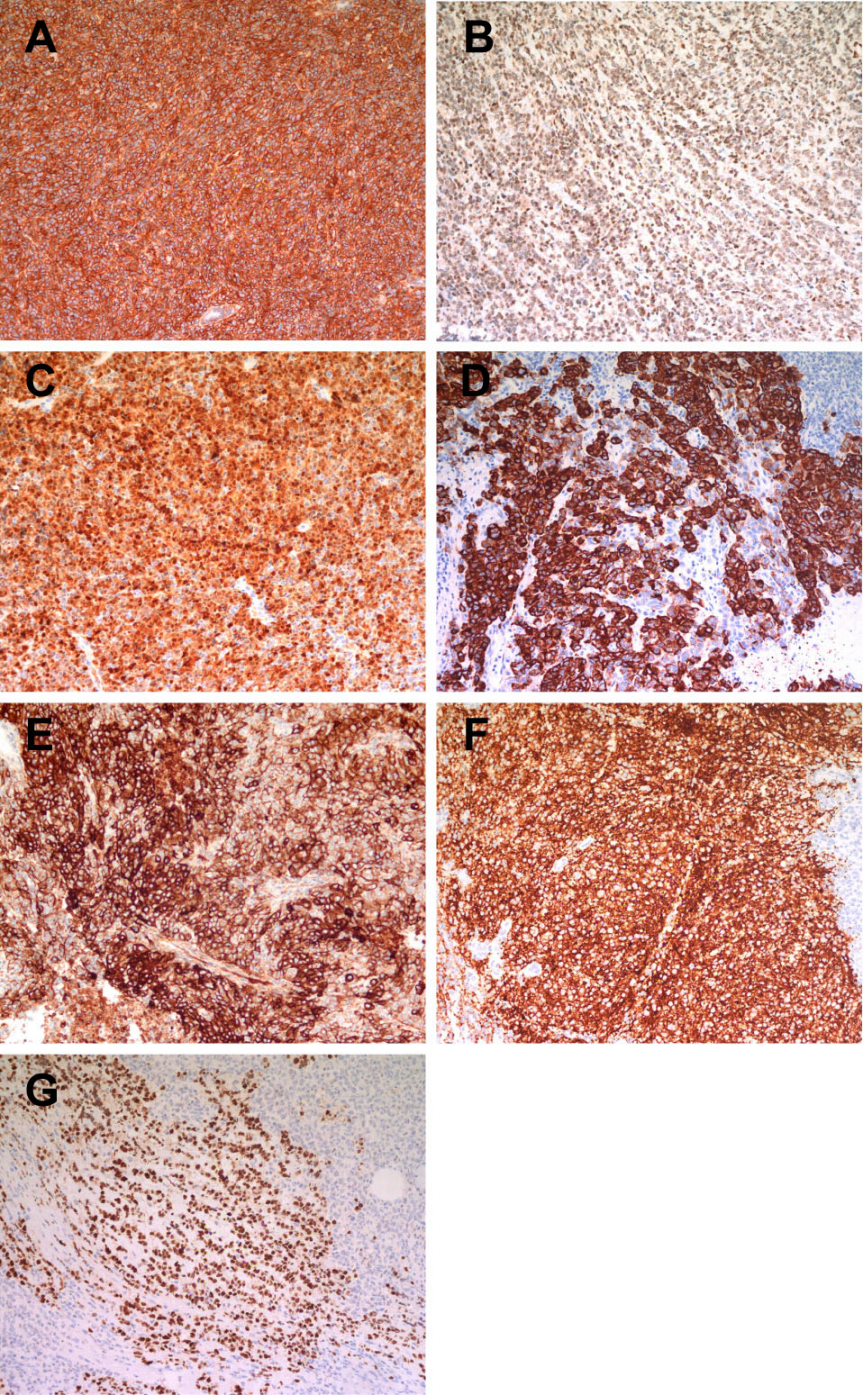
**(A-C)**: Immunohistochemical staining for CD99, Fli1, and S-100, respectively. The undifferentiated round cells show diffuse positivity for CD99, weak positivity for Fli1, and S-100 exhibits focal staining across all three differentiation regions. **(D, E)** The epithelioid cells demonstrate diffuse expression of PCK and EMA, respectively. **(F, G)** The neural differentiation areas are positive for synaptophysin, and the small round cells within these regions show positivity for NKX2.2.

**Figure 4:**
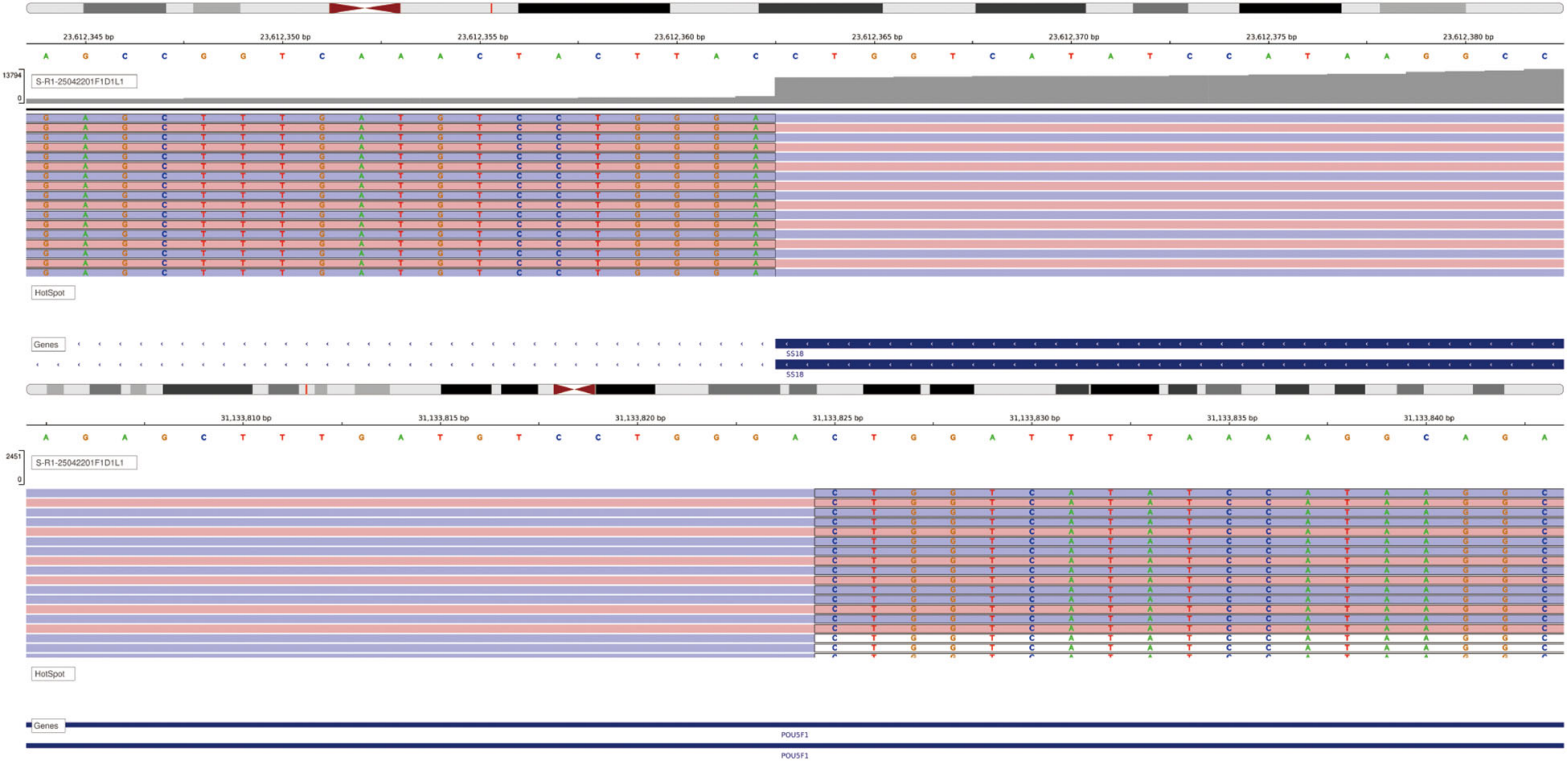
NGS identified an in-frame fusion transcript resulting from the junction between exon 10 of the SS18 gene and exon 2 of the POU5F1 gene.

**Figure 5:**
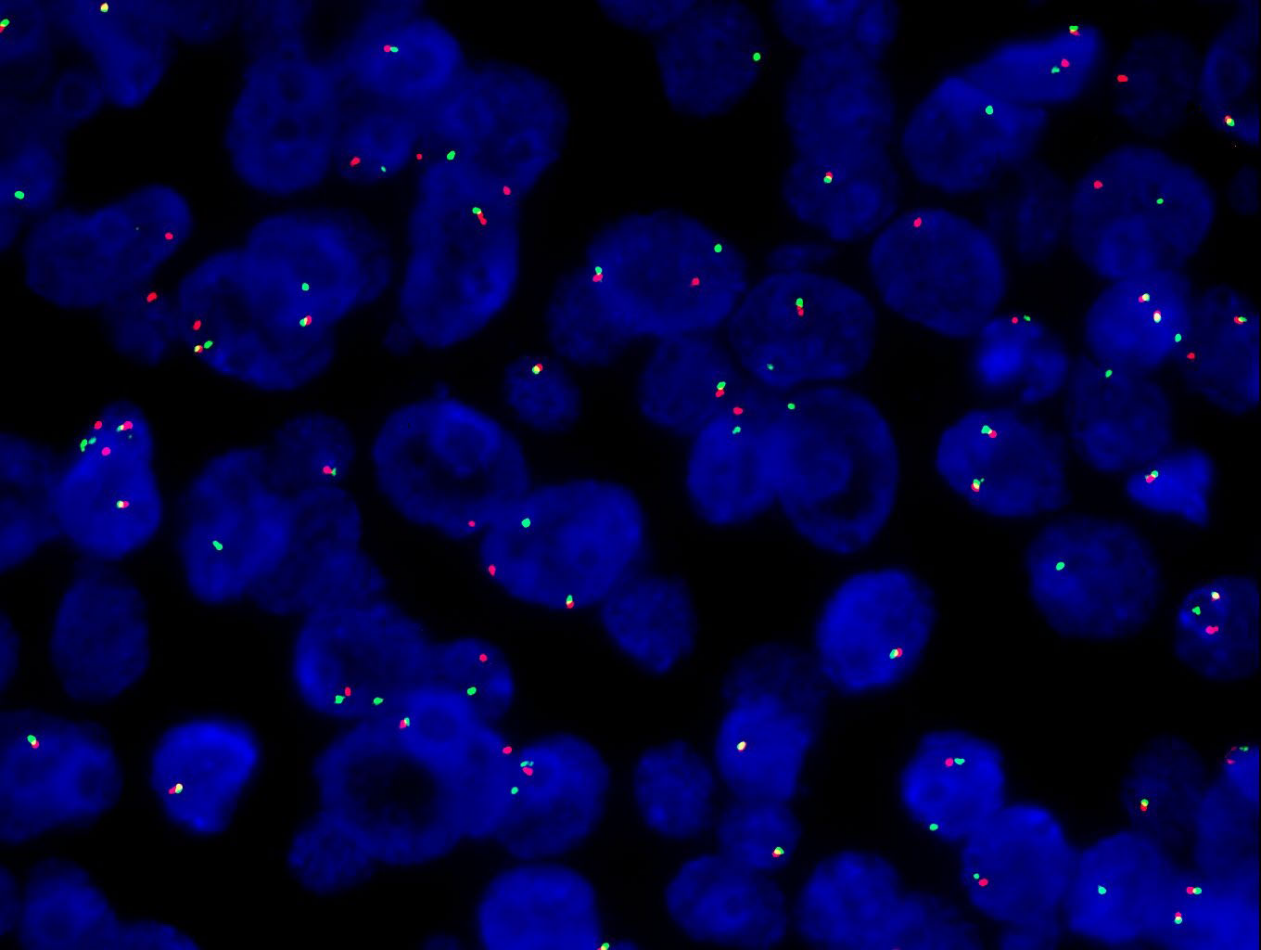
SS18 break-apart probe demonstrates a split signal pattern.

The specimen is a nodular mass measuring 5.0 cm × 3.8 cm × 2.0 cm, with a partial capsule and attached skeletal muscle fragments at the periphery. The cut surface appears tan-white to red and is soft in consistency. Histologically, the tumor demonstrates a sheet-like and diffuse growth pattern with focal fibrous pseudocapsule formation. It exhibits considerable morphological heterogeneity. Approximately 90% of the tumor cells consist of diffusely arranged, undifferentiated small round cells with scant amphophilic cytoplasm and round nuclei. Some nuclei display irregular nuclear membranes, finely stippled chromatin, and small nucleoli. Mitotic figures are readily identifiable.The remaining 10% of tumor cells are larger in size, featuring prominent nucleoli and abundant, pale eosinophilic cytoplasm. These cells are arranged in nested patterns, exhibiting an epithelioid morphology (**Figures 2A–E**). Focal papillary structures formed by tumor cells surrounding blood vessels are observed, accompanied by stromal mucinous degeneration (**Figure 2F**). Areas indicative of neural differentiation are present, characterized by a neuropil-like background and the formation of Flexner-Wintersteiner rosettes (**Figures 2G, H**). Residual salivary gland tissue is identified within the tumor, with evident invasion into adjacent skeletal muscle. IHC staining was performed using Roche and Dako automated platforms (BenchMark XT, Autostainer Link 48) with the EnVision two-step method. Antibodies included PCK, CK5/6, P40, CK7, CD99, FLI1, NKX2.2, EMA, S-100, SOX10, Syn, Calretinin, PHOX2B, INI1, BRG1, H3K27Me3, P53, SS18-SSX, ERG, CD34, SMA, Desmin, WT1, BCOR, NUT and Ki67. The undifferentiated round cell component exhibited diffuse positivity for CD99, weak positivity for FLI1, and partial positivity for S-100 and NKX2.2, while being negative for EMA and PCK. The epithelioid areas demonstrated diffuse expression of PCK and EMA, partial positivity for S-100, and were negative for CD99, NKX2.2, and FLI1. The region with neural differentiation showed expression of Syn, NKX2.2, and S-100. No ganglion cell component was detected with Calretinin or PHOX2B staining. No loss of expression was observed for INI1, BRG1, or H3K27Me3. Approximately half of the tumor cells exhibited heterogeneous weak-to-strong staining for P53, suggesting a wild-type expression pattern. All other markers—including CK5/6, P40, CK7, SOX10, SS18-SSX, ERG, CD34, SMA, Desmin, WT1, BCOR, and NUT—were negative. The Ki67 proliferation index was approximately 40% (**Figure 3**). Based on the above findings, we have two preliminary considerations for the pathological diagnosis of this case: extraskeletal Ewing sarcoma or round cell sarcoma with EWSR1-non-ETS family gene fusion. However, the Next-generation sequencing (NGS) analysis revealed a fusion transcript formed by the junction of exon 10 of the SS18 gene and exon 2 of the POU5F1 gene (**Figure 4**). Fluorescence *in situ* hybridization (FISH) using SS18 break-apart probes demonstrated a separation signal pattern in tumor cells across all differentiated regions (**Figure 5**), confirming the presence of an SS18 gene rearrangement.

Subsequently, the patient received two cycles of postoperative adjuvant chemotherapy with the VDC regimen (vincristine, doxorubicin, and cyclophosphamide) and one cycle of IE regimen (ifosfamide and etoposide) chemotherapy, all in combination with serplulimab immunotherapy. Half a month after the initial surgery, contrast-enhanced MRI of the neck and maxillofacial region revealed a recurrent mass at the primary tumor bed. Following three cycles of combined chemotherapy and immunotherapy, follow-up imaging demonstrated significant reduction in two newly developed lesions in the left submandibular region. The cross-sectional dimensions of the lesions decreased from 17 mm × 19 mm × 13 mm and 44 mm × 19 mm × 19 mm to 8.7 mm × 8.1 mm × 11 mm and 9 mm × 7 mm × 17 mm, respectively.

On July 3, 2025, the patient underwent left submandibular lesion resection with functional neck dissection. Postoperative pathological examination confirmed tumor recurrence. Microscopically, the tumor cells predominantly exhibited an epithelioid morphology, consistent with the cellular features and immunophenotype of the epithelioid-differentiated areas in the originally resected specimen. The proportion of undifferentiated small round cell components was less than 5%, with no evidence of neural differentiation. No tumor metastasis was detected in the surrounding lymph nodes. Although the postoperative follow-up period is relatively short (6 months since the second surgery), the patient remains under close surveillance, and to date, no evidence of tumor recurrence or metastasis has been observed.

## Discussion

A systematic search of the PubMed database yielded only Six previously published cases of SS18::POU5F1-fused sarcoma in the English literature, underscoring the extreme rarity of this entity (1–5). The ages of the reported patients ranged from 11 to 40 years (4 females and 2 males), with tumors occurring in soft tissue (3 cases), kidney, pancreas, and parotid gland. Morphologically, all tumors contained an undifferentiated round cell component, arranged in sheets, nests, or trabeculae; focal epithelioid morphology was observed in some cases. One case showed tumor cells with rhabdoid/rhabdomyoblastic differentiation (4), and another exhibited ganglioneuroma-like differentiation (5). The tumor cells generally exhibited vesicular nuclei with variably prominent nucleoli and readily identifiable mitotic figures. All cases were positive for EMA, and at least focal expression of PCK and S100 was observed (5/6), suggesting a possible myoepithelial immunophenotype (**Table 1**). Myogenic, vascular, and melanocytic markers were negative in all cases. No loss of INI1 or BRG1 was detected in any of the cases examined. The clinical features, histology, and immunohistochemical profiles of these previously reported cases and the present case are summarized in **Table 1**.

**Table 1:** Clinicopathological and molecular features of cases of SS18::POU5F1-fused sarcomas (1–5).

Author	Age/ gender	Site	Outcome	Histology	IHC (+)	IHC (–)	Molecular findings
Antonescu 2020.case 1 (1)	18,M	Pancreas with Liver metastases	DOD 31 months (systemic metastases)	Small round cell/epithelioid	EMA (diffuse); AE1/3, Cam5.2, S100, TLE1, CD99 (all focal), Synaptophysin (rare). Ki67 90%	P40, p63, S100, SOX10, HMB45, desmin, WT-1, GATA3, chromogranin, hCG, INSM-1, hPL, trypsin, chymotrypsin, PR, OCT4, CD30, SALL4.	SS18 (exon 10)::POU5F1 (exon 2)
Antonescu 2020.case2 (1)	29,F	Subcutaneous mass	NED 10 months with adjuvant chemotherapy	Small round cell/epithelioid	S100 (focal), EMA(weak focal)	Pan Cytokeratin, CK7, CK20, CK8/18, SOX10, HMB45, melan A, WT1, CD117, CD99, TLE1, STAT6, BCOR, desmin, myogenin, synaptophysin, TTF1, PLAP, HCG, ER, CD3, CD20, CD30, CD31, CD34, CD43, MUM1, MPO, TdT. INI1, BRG1, BRM1 retained.	SS18 (exon 10)::POU5F1 (exon 2)
Shenoy 2020 (2)	11,F	Subcutaneous mass with LN metastasis	Unknown	Small round cell/epithelioid	AE1/3, EMA, S100 (diffuse)	CD99, desmin, myogenin, WT-1, ERG, CD45, SOX10, HMB45, p63, GFAP, calponin, TLE1. INI1 retained.	SS18 (exon 10)::POU5F1 (exon 2)
Liu 2021 (3)	18,F	Soft tissue mass with LN metastases	DOD 8 months (systemic metastases)	Small round cell/epithelioid	Pan Keratin, EMA, CD31, BCl2, vimentin, CD99, S100 (focal)	CD34, HMB45, MyoD1, myogenin, ERG, Factor 8.	SS18 (exon 9)::POU5F1 (exon 2)
Argani 2022 (4)	38,M	Kidney	Unknown	Small round cell/epithelioid/rhabdoid/spindle	EMA, PAX8 (diffuse); S100, TLE1 (patchy); AE1/3 (focal)	GATA3, Ck7, CK20, WT1, desmin, actin, CD99, NKX2.2, Oct3/4. INI1, BRG1 retained.	SS18 (exon 10)::POU5F1 (exon 2)
Ming Liang Oon 2025 (5)	40,F	Parotid mass with LN metastasis	NED 6 months	Predominantly epithelioid cells /GN component	CK, EMA (diffuse); S100, GFAP, Calretinin (GN component)	CD99, P63, SS18-SSX, PHOX2B, NeuN, EBER-ISH, Desmin, CD30, OCT4, AR, Her2.	SS18 (exon 10)::POU5F1 (exon 2)
Our case	38,M	Submandibular gland mass with LN metastasis	NED 3 months	Small round cell/epithelioid/neural differentiation	Small round cell: CD99 (diffuse), FLI1 (weak), NKX2.2(patchy); epithelioid: PCK, EMA (diffuse); neural differentiation: syn, NKX2.2; S-100 (patchy), Ki67 (LI:40%).	Calretinin, PHOX2B, P53, CK5/6, P40, CK7, SOX10, SS18-SSX, ERG, CD34, SMA, Desmin, WT1, BCOR, NUT. INI1, BRG1, H3K27Me3 retained.	SS18 (exon 10)::POU5F1 (exon 2)

LN, lymph node; DoD, dead of disease; NED, no evidence of disease; GN, ganglioneuromatous.

We describe a case of SS18::POU5F1-fused sarcoma arising in the left submandibular gland of a 38-year-old male, notable for the presence of Flexner-Wintersteiner rosettes—a finding not previously reported in this tumor type. While its morphological and immunohistochemical features show similarities to previously reported cases, it also demonstrates several unique characteristics. Histologically, the tumor predominantly grew in solid sheets and nests, with focal formation of broad papillary-like structures around blood vessels—a feature not previously documented. The tumor was composed mainly of undifferentiated round cells within a richly vascularized stroma, histologically resembling Ewing sarcoma or round cell sarcoma with EWSR1::non-ETS fusion. Focal neural differentiation was observed, characterized by a neuropil-like background and rosette formations. These rosettes were identified as Flexner-Wintersteiner rosettes, which have not been previously reported in this tumor type. In contrast to the ganglioneuromatous differentiation described by Oon et al. (5), the present case exhibited neural differentiation solely in the form of a neuropil-like background and Flexner-Wintersteiner rosettes; no ganglion cells were observed. Despite documented cases of Ewing family tumors exhibiting ganglioneuromatous or gangliogliomatous differentiation (6, 7) (one of which harbored an EWSR1::POU5F1 fusion (6)), a clear correlation between this morphological pattern and specific genetic alterations has not been established.

Among the reported cases, five out of six exhibited a fusion between exon 10 of SS18 and exon 2 of POU5F1, while one case involved a fusion between exon 9 of SS18 and exon 2 of POU5F1 (3). In our case, NGS identified an SS18 (exon 10)::POU5F1 (exon 2) fusion gene (**Figure 3**), consistent with the majority of reported cases. FISH using SS18 break-apart probes revealed a split signal pattern (**Figure 4**), confirming rearrangement of the SS18 gene.

The nature of SS18::POU5F1-fused sarcoma remains controversial, with ongoing debate as to whether it represents a molecular variant of synovial sarcoma, a malignant myoepithelial tumor, or a distinct type of round cell sarcoma. The seminal study by Antonescu et al. (1) demonstrated that SS18::POU5F1 fusion sarcomas cluster molecularly with myoepithelial tumors harboring EWSR1/FUS::POU5F1 fusions, but not with synovial sarcomas carrying SS18 rearrangements. Notably, however, these tumors also did not cluster with myoepithelial tumors bearing non-POU5F1 fusions (such as EWSR1::ZNF44 or FUS::KLF17).

All previously reported cases were positive for EMA, and the majority showed at least focal expression of PCK and S100 (5/6). Although non-specific, this immunoprofile suggests a possible myoepithelial phenotype. In our case, a subset of tumor cells exhibited epithelioid morphology with abundant pale cytoplasm, large nuclei, and prominent nucleoli, arranged in solid sheets and nests, morphologically resembling a myoepithelial tumor. These areas demonstrated diffuse expression of PCK and EMA, partial positivity for S100, and were negative for other epithelial and myoepithelial markers. Although a single case may not be representative, these findings do not provide conclusive evidence for definitive myoepithelial differentiation. Further studies are warranted to elucidate the biological origin of this tumor type.

The POU5F1 gene encodes the transcription factor OCT-4, a key regulator of cellular totipotency (8, 9) that is also expressed in adult stem cells (10). Overexpression of POU5F1 can trigger epithelial-mesenchymal transition (EMT) (9), a process linked to tumor transdifferentiation (11), including in sarcomatoid carcinoma (12). Antonescu et al. hypothesized that the diverse cell types observed in soft tissue myoepithelial tumors with EWSR1::POU5F1 fusions may result from multiphenotypic differentiation (13, 14).

SS18 is a specific subunit of the BRG/BRM-associated factor (BAF) complex (15) and has been shown to regulate the transition from pluripotent to somatic cell states. Studies indicate that loss of SS18 delays the differentiation of pluripotent cells into somatic lineages (16). Although SS18 is most commonly associated with synovial sarcoma, recent reports have described round cell undifferentiated sarcomas with CRTC1::SS18 fusions (15, 17, 18). Sarcomas with CRTC1::SS18 fusion are morphologically and immunophenotypically distinct from those with SS18::POU5F1 fusion (18). The former often exhibit stromal hyperplastic areas and grooved tumor nuclei, and are immunopositive for CD99, negative for S100, and mostly negative for keratins. Notably, since both fusion partners—SS18 and POU5F1—are involved in regulating cell differentiation, it is plausible that the biphasic or multidirectional differentiation observed in SS18::POU5F1-fused sarcomas may be driven by this genetic alteration.

The clinical, radiological, and histological features of SS18::POU5F1-fused sarcoma frequently prompt a broad differential diagnosis, most notably including Ewing sarcoma, synovial sarcoma, and myoepithelial tumors. A definitive diagnosis can only be rendered after a comprehensive integration of histomorphology, immunophenotype, and—most critically—molecular findings. In the present case, the submandibular location and imaging findings were non-specific and did not reliably distinguish among these entities, further underscoring the essential role of integrated pathological and molecular evaluation. As summarized in **Table 2**, comparing the characteristic morphological, immunohistochemical, and molecular profiles of SS18::POU5F1-fused sarcoma with its principal mimics clearly reveals the comprehensive basis for precisely defining this entity. This reiterates that the exclusion of these differential diagnoses does not rely on any single indicator, but is founded on the integrated interpretation of morphological, immunohistochemical, and, above all, molecular data.

**Table 2:** Key points for the differential diagnosis of SS18-POU5F1 fusion sarcoma.

Tumor type	SS18::POU5F1-fused sarcoma	Ewing sarcoma	Synovial sarcoma	Myoepithelial tumor/carcinoma
Location	• Internal organs • Soft tissue • Salivary glands	• Bone • Soft tissues	• Extremities • Head and neck (<10%)	Salivary glands, especially the parotid
Morphologic Features	• Small round cell/epithelioid • Rhabdoid/spindle • GN component • Flexner-Wintersteiner rosettes	• Monotonous small round blue cells •Homer-Wright rosettes	• Biphasic: Epithelioid and spindle cell • Monophasic: spindle cells	Wide morphologic spectrum: • Plasmacytoid, spindle, epithelioid, or clear cells • Solid sheets, nests, or reticular patterns
Immunohistochemistry	• EMA+ • CK,S100 (focal) • CD99+/- • NKX2.2+(neural differentiation)	• CD99+ • NKX2.2+ • FLI-1+ • CK, EMA -	• CK, EMA+(epithelioid and some spindle cells) • SS18-SSX, TLE1+ • BCL-2+ • CD99+	• CK+ • p63, p40, Calponin, SMA, S100, SOX10+
Molecular Alteration	SS18::POU5F1	• EWSR1::FLI1 • EWSR1::ERG	SS18::SSX (SSX1/SSX2/SSX4)	Heterogeneous genetic alterations, PLAG1 or HMGA2 rearrangements

Due to the rarity of tumors with SS18::POU5F1 fusion, their clinical behavior, optimal treatment, and prognosis remain poorly defined. Liu et al. (3) reported an 18-year-old female with SS18::POU5F1 fusion sarcoma in the left inguinal region, accompanied by mediastinal, subclavian, and pelvic lymphadenopathy. Following tumor resection, the patient received adjuvant therapy with anlotinib, cetuximab, and radiotherapy, achieving a partial response. However, intra-abdominal progression occurred within six weeks during treatment, prompting chemotherapy with doxorubicin and ifosfamide. This suggests that the tumor may exhibit a rapid, robust, yet transient response to radiotherapy.

In our case, the patient received three cycles of chemotherapy combined with immunotherapy after surgery. Imaging revealed significant shrinkage of two new submandibular lesions: the cross-sectional dimensions decreased from 17mm × 19mm × 13mm and 44mm × 19mm × 19mm to 8.7mm × 8.1mm × 11mm and 9mm × 7mm × 17mm, respectively, indicating a favorable response to the chemoimmunotherapy regimen. Nonetheless, tumor recurrence occurred within half a month after surgery, suggesting highly aggressive behavior. The recurrent tumor consisted predominantly of epithelioid cells with an immunophenotype consistent with the initial resection specimen. The proportion of undifferentiated small round cell components was less than 5%, and no neural differentiation was observed, implying that the epithelioid component may be associated with greater invasiveness.

It is important to note that the current follow-up period remains short, and the patient is under close surveillance. Given the extreme rarity of reported cases and limited clinical experience with this tumor entity, longer-term follow-up is essential to fully understand its clinical course, treatment response patterns, and long-term outcomes. Continued accumulation of clinical data will be critical to inform optimal treatment strategies for patients with SS18::POU5F1-fused sarcoma.

## Conclusion

The widespread application of sequencing technologies in clinical practice has enabled molecular characterization of an increasing number of undifferentiated tumors, revealing the limitations of defining tumor entities based solely on morphological or genomic features. This study presents the first reported case of a malignant round cell tumor occurring in the submandibular gland, exhibiting a Ewing-like undifferentiated morphology, epithelioid tumor cells, and Flexner-Wintersteiner rosettes, harboring an SS18::POU5F1 fusion. The epithelioid component within this tumor appears to demonstrate heightened aggressiveness. While such tumors display an immunophenotype overlapping with Ewing sarcoma or myoepithelial tumors, their fundamental nature—whether they represent a molecular subtype of synovial sarcoma, a malignant myoepithelial tumor, or a distinct type of round cell sarcoma—remains controversial. Further research is needed to explore the fusion-driven oncogenic mechanisms and elucidate the downstream effects of this fusion gene to clarify its biological identity. However, due to the extreme rarity of these cases, the clinical behavior and optimal treatment strategies for SS18::POU5F1 fusion tumors remain uncertain, necessitating continued accumulation of clinical experience and data.

## Data Availability

The original contributions presented in the study are included in the article/supplementary material. Further inquiries can be directed to the corresponding author.
